# *ARHGAP35* is a novel factor disrupted in human developmental eye phenotypes

**DOI:** 10.1038/s41431-022-01246-z

**Published:** 2022-12-01

**Authors:** Linda M. Reis, Nicolas Chassaing, Tanya Bardakjian, Samuel Thompson, Adele Schneider, Elena V. Semina

**Affiliations:** 1 Department of Pediatrics and Children’s Research Institute, Medical College of Wisconsin and Children’s Wisconsin, Milwaukee, WI USA; 2 Service de Génétique Médicale, Hôpital Purpan CHU Toulouse, Toulouse, France; 3 Platerforme AURAGEN, Lyon, France; 4 Einstein Medical Center Philadelphia, Philadelphia, PA USA; 5 Department of Ophthalmology and Visual Sciences, Medical College of Wisconsin, Milwaukee, WI USA

**Keywords:** Genetics research, Development

## Abstract

ARHGAP35 has known roles in cell migration, invasion and division, neuronal morphogenesis, and gene/mRNA regulation; prior studies indicate a role in cancer in humans and in the developing eyes, neural tissue, and renal structures in mice. We identified damaging variants in *ARHGAP35* in five individuals from four families affected with anophthalmia, microphthalmia, coloboma and/or anterior segment dysgenesis disorders, together with variable non-ocular phenotypes in some families including renal, neurological, or cardiac anomalies. Three variants affected the extreme C-terminus of the protein, with two resulting in a frameshift and C-terminal extension and the other a missense change in the Rho-GAP domain; the fourth (nonsense) variant affected the middle of the gene and is the only allele predicted to undergo nonsense-mediated decay. This study implicates *ARHGAP35* in human developmental eye phenotypes. C-terminal clustering of the identified alleles indicates a possible common mechanism for ocular disease but requires further studies.

## Introduction

Developmental ocular disorders including microphthalmia, anophthalmia, and coloboma (MAC) and anterior segment dysgenesis (ASD) spectrums represent an important cause of vision loss in childhood. Genetic diagnoses are unable to be established in a significant portion of cases: 70–85% of MAC and 40–75% of ASD cases remain unexplained after genetic analysis according to recent studies [1–4]. While some have idiopathic or environmental causes, others are likely caused by pathogenic variants in genes with a currently unrecognized role in eye development. Establishing a genetic diagnosis is important for clinical management of affected children and provides other family members with the opportunity to clarify their genetic status/risk. Identification of novel genetic causes of ocular disorders enhances our understanding of the mechanisms of ocular development, generating additional opportunities for therapeutic intervention in the future.

ARHGAP35 (Rho GTPase Activating Protein 35), also known as GRLF1 (Glucocorticoid Receptor DNA-binding Factor 1) or p190ARhoGAP-A, is a GTPase-activating protein that regulates GTPases within the Rho and Rac families [5, 6]. It has known direct roles in cell migration/invasion and division as well as neuronal morphogenesis and dendritic spine formation; additional roles include gene/mRNA translation regulation through interaction with TFII-I and eiF3A [5]. Mice homozygous for a loss-of-function *Arhgap35* allele display highly penetrant early lethality, structural brain anomalies, cystic glomeruli, and optic cup anomalies including coloboma and microphthalmia while heterozygous mice are unaffected; a subset of homozygous animals also display neural tube closure defects, particularly exencephaly [7, 8]. Little is known about the role of germline variants in humans; only one de novo missense variant, c.1801G > T p.(Val601Phe), has been reported in the literature: limited phenotypic description reported a terminated pregnancy with severe midline birth defects [9]. The rate of de novo variants in *ARHGAP35* was also noted to be enriched in a large cohort of individuals with developmental disorders with no details provided [10]. Through exome/genome sequencing and GeneMatcher, we identified a cohort of individuals with developmental ocular disorders and novel damaging variants in *ARHGAP35*.

## Materials and methods

This human study was approved by Institutional Review Boards at Children’s Wisconsin and Einstein Medical Center Philadelphia. Written informed consent including research analysis and photo publication if applicable was obtained for every participant. Exome sequencing was undertaken by Psomagen (Rockville, MD) and analyzed with VarSeq (Golden Helix, Bozeman, MT). In silico analysis of variants of interest included filtering for frequency <0.001 in the general population in gnomAD v2.1.1 [11] and for predicted effect upon the protein. The effect of missense variants on protein function was further analyzed by two combined analysis tools (CADD phred hg19 and REVEL). Samples were first analyzed for variants in known MAC and ASD genes as previously described [12, 13]. Trio analysis in negative cases identified *ARHGAP35* as a candidate in two families and screening for variants in this gene specifically identified one more case. Sanger sequencing was used to confirm variants and for segregation analysis. An additional case was identified through clinical genome sequencing and Matchmaker Exchange Databases [14]. Variants in *ARHGAP35* were named based on reference sequence NM_004491.4 and human Genome Build hg19 and evaluated according to ACMG/AMP guidelines [15].

## Results

Novel damaging variants in *ARHGAP35* were identified in five individuals with developmental ocular disorders from four families. Pathogenic variants in other MAC/ASD genes were ruled out in all individuals.

**Fig. 1:**
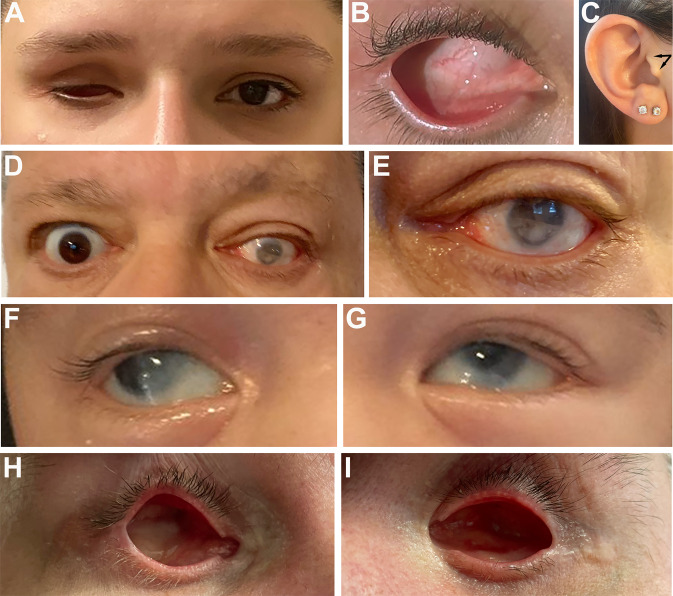
Ocular images from individuals with *ARHGAP35* variants. **A**–**C** Images from Individual 1A showing right eye severe microphthalmia and sclerocornea, normal left eye, and bifid tragus. **D**, **E** Images from Individual 1B showing left eye coloboma and microcornea/microphthalmia and normal right eye. **F**, **G** Images from Individual 2 showing bilateral Peters anomaly. **H**, **I** Images from Individual 4 showing bilateral anophthalmia.

**Fig. 2:**
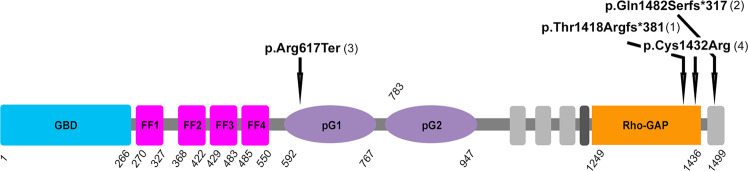
ARHGAP35 protein structure. Structure of ARHGAP35 protein (per Uniprot Q9NRY4) with variant positions indicated. Family number for each variant is given in parentheses after the variant. GBD: (GTP)-binding domain, FF: FF domains, pG1&2: pseudoGTPase domains, Rho-GAP: GTPase activating protein domain, dark gray unlabeled box: region required for phospholipid binding and regulation of the substrate preference; light gray unlabeled boxes: disordered regions.

Individual 1A is a 27-year-old female born with right ocular anomalies consisting of severe microphthalmia (axial length 7.6 mm) and sclerocornea; her left eye is normal (Fig. 1A, B). She does have a bifid tragus on both ears. Her father, Individual 1B, is a 68-year-old male born with left ocular anomalies and esotropia; his right eye is normal (Fig. 1C, D). He was diagnosed with left mild microphthalmia with iris and chorioretinal coloboma and a localized spoke shaped opacity in the lens at 6 o’clock at 6 months of age. Additional features included a hemangioma on the right forearm and history of cancer (histocyte rich B cell non-Hodgkin’s lymphoma at 38 years of age and Nodular Lymphocyte Predominant Hodgkin Lymphoma at 61 years of age). Growth and development were normal for both individuals. Exome sequencing identified a heterozygous novel variant in *ARHGAP35* c.4251delC p.(Thr1418Argfs*381) shared by both affected individuals. This variant was not present in five unaffected family members: mother, brother, paternal grandmother, and two paternal aunts (paternal grandfather unavailable) (Table 1, Fig. 2, Supplementary Fig. 1A). This variant meets ACMG criteria to be considered Pathogenic (PVS1, PM2_supporting, PP1).

**Table 1:** Summary of *ARHGAP35* variant and clinical data.

Case number	Individual 1A	Individual 1B	Individual 2	Individual 3	Individual 4A	Individual 4B
Inheritance	AD	Father of 1A	de novo	de novo	Inherited	Father of 4A
Location (hg19)	19:47503695-C-	19:47503695-C-	19:47503884-C-	19:47423781-C-T	19:47503739-T-C	19:47503739-T-C
Nucleotide^a^	c.4251delC	c.4251delC	c.4444delC	c.1849C > T	c.4294 T > C	c.4294 T > C
Protein	p.(Thr1418Argfs*381)	p.(Thr1418Argfs*381)	p.(Gln1482Serfs*317)	p.(Arg617*)	p.(Cys1432Arg)	p.(Cys1432Arg)
Location	End of Rho-GAP domain	End of Rho-GAP domain	C-terminal to Rho-GAP domain	pG1 domain	End of Rho-GAP domain	End of Rho-GAP domain
Predicted effect	Truncation of normal protein in final exon and erroneous tail extension	Truncation of normal protein in final exon and erroneous tail extension	Truncation of normal protein in final exon and erroneous tail extension	Truncation in first exon	Missense in Rho-GAP domain	Missense in Rho-GAP domain
CADD score	N/A	N/A	N/A	N/A	32	32
REVEL score	N/A	N/A	N/A	N/A	0.439	0.439
gnomAD^b^	NP	NP	NP	NP	NP	NP
Read depth NGS	113/270 (42%)	119/228 (52%)	9/27 (33%)	16/28 (57%)	90/160 (56%)	68/184 (37%)
Sanger confirmed	Yes	Yes	Yes	No	Yes	Yes
ACMG criteria	P: PVS1, PM2_supp, PP1	P: PVS1, PM2_supp, PP1	P: PVS1, PS2, PM2_supp	P: PVS1, PS2, PM2_supp	VUS: PM2, PP2, PP3	VUS: PM2, PP2, PP3
Gender	Female	Male	Male	Male	Male	Male
Age (y)	27	68	2	3	42	U
Ocular	R severe microphthalmia with sclerocornea	L inferior iris, choroidal coloboma, cataract, microphthalmia, uveitis (30 y), retinal detachment; R diabetic retinopathy only	B Peters anomaly (type II) treated with k-pro	R microphthalmia, sclerocornea; L microphthalmia, iris hypoplasia, corectopia, B agenesis of optic nerves	B anophthalmia	Glasses from a young age but no MAC
Craniofacial	B bifid tragus	–	Nevus flammeus of the glabella	–	–	–
Development	WNL	WNL	WNL	Delayed: Nonambulatory at 27 m	WNL	WNL
Cardiac	–	–	Pulmonary valve stenosis, thickened aortic leaflet	–	–	–
Renal	–	–	Large L kidney with possible duplex anatomy	L duplicated ureters	–	–
Cancer	–	Lymphoma at 38 y and 61 y	-	–	–	–
Other	–	Type 2 IDDM; Hemangioma on R forearm	Nuchal cord at birth; Small capillary hemangioma on occiput	Macrocephaly likely related to a de novo *PTEN* variant chr10: 89624228 T > G	–	–

*NP* not present, *P* Pathogenic, *VUS* variant of uncertain significance, *B* bilateral, *L* left, *R* right, *N/A* not applicable, *WNL* within normal limits, *m* months, *y* years.

^a^NM_004491.4.

^b^gnomADv2.1.1.

Individual 2 is a 2-year-old male with bilateral type II Peters anomaly consisting of corneal opacity with cataract, iris hypoplasia, and glaucoma treated with keratoprostheses (Fig. 1E, F). Additional features included pulmonary stenosis and thickened aortic leaflet, large left kidney with possible duplex anatomy, nevus flammeus of the glabella, small capillary hemangioma on the occiput, and nuchal cord at birth. He has had normal growth and development to date. Trio exome sequencing of the child and unaffected parents identified a de novo novel variant in *ARHGAP35*, c.4444delC p.(Gln1482Serfs*317) (Table 1, Fig. 2, Supplementary Fig. 1B). This variant meets ACMG criteria to be considered Pathogenic (PVS1, PS2, PM2_supporting).

Individual 3 is a 3-year-old male who presented with bilateral microphthalmia (axial lengths 13.65 mm and 14.31 mm) and agenesis of the optic nerves. Sclerocornea was observed in the right eye while the left had iris hypoplasia and corectopia. Growth was normal but development showed hypotonia and significant delay (non-ambulatory at 27 months). Additional features include left duplicated ureters and macrocephaly (55 cm at 27 weeks, +4.55 SD). Echocardiogram, skeletal survey, and Brain MRI were normal other than eye and optic nerve anomalies. Pregnancy history is notable for increased nuchal translucency at 12 weeks gestation and identification of renal anomalies and macrocephaly at 22 weeks gestation. Trio genome sequencing of the child and unaffected parents identified de novo novel variants in *PTEN*, NM_000314.8:c.2 T > G p.(Met1Arg), and *ARHGAP35*, c.1849C > T p.(Arg617Ter) (Table 1, Fig. 2, Supplementary Fig. 1C). This variant meets ACMG criteria to be considered Pathogenic (PVS1, PS2, PM2_supporting).

Individual 4 is a 42-year-old male with bilateral anophthalmia. Trio exome sequencing identified a novel variant in *ARHGAP35*, c.4294 T > C p.(Cys1432Arg), inherited from the father, who did not have a MAC phenotype but was reported to wear glasses from a young age with no further details available (Table 1, Fig. 2, Supplementary Fig. 1D). The exome read depth was slightly skewed in the father (37%) but Sanger sequencing showed even peaks (Supplementary Fig. 1D). This missense variant is predicted to be damaging with high CADD (32) and GERP + + (5.6) scores and a moderate REVEL score (0.439). This variant is considered a Variant of Uncertain Significance by ACMG criteria (PM2, PP2, PP3).

## Discussion

This study presents the first evidence implicating *ARHGAP35* in human developmental ocular phenotypes. Three variants affected the extreme C-terminus of the protein, with two resulting in a frameshift and C-terminal extension and the other a missense change in the Rho-GAP domain. General population data in gnomAD shows significant constraint against both loss of function and missense variants for this gene [11]. The MAC phenotypes observed here are very consistent with those reported in the mouse model. ARHGAP35 was also previously shown to be involved in the regulation of genes within the Hippo signaling pathway [16] including *YAP1*, which is independently associated with MAC disorders [17–19]. Corneal defects, observed in three families in this study, are also seen in *Yap1-*deficient mice [20].

The non-ocular features within our cohort were more variable. The presence of duplicated renal structures in two individuals is intriguing, though it does not perfectly replicate the hypodysplasia phenotype observed in mice. With regards to the history of cancer in Individual 1B, while a tumor suppressor role has been identified for ARHGAP35, particularly in carcinomas [5], an association with lymphoma has not been reported to date so it is unclear whether this phenotype is coincidental or related to the genetic variant. Interestingly, only one variant was associated with an additional neurological phenotype in this cohort (Individual 3, p.(Arg617Ter)). That variant occurred earlier in the gene, within the first exon, and is expected to lead to complete loss of function due to nonsense-mediated decay. Mice with neurological phenotypes similarly had complete loss-of-function alleles [7, 8]. The other three variants identified in individuals with normal neurological function occurred in or after the RhoGAP domain in the final exon of the gene; thus, the variant proteins would be expected to escape nonsense-mediated decay and may retain enough function for normal neurological development. However, the patient with a neurological phenotype also has a *PTEN* variant in the initiation codon. While similar *PTEN* variants have been reported in individuals with macrocephaly and variable autism/intellectual disability [21], the hypotonia/gross motor phenotype is more severe than is typical for *PTEN* variants, so it is likely that the *ARHGAP35* variant is also contributing to the neurological phenotype.

Two of the four variants were de novo, supporting a likely causative role. One of the others was inherited from an affected parent- while it could not be conclusively proven to be de novo, it was not present in five unaffected family members. Interestingly, in this family both affected individuals had a unilateral ocular phenotype, similar to the recently reported *PRR12* gene with dominant unilateral MAC or ASD phenotypes [22]. In the final case, the variant was inherited from a parent without MAC but with reduced vision that required corrective lenses. Because available clinical data was limited, it is impossible to conclude whether this represents a case of incomplete penetrance, variable expressivity, or somatic mosaicism (though no strong evidence of mosaicism was detected); it is also possible that this is a benign allele that does not contribute to the ocular phenotype. Incomplete penetrance and variable expressivity are noted for other MAC genes including *YAP1* (regulated by ARHGAP35).

In combination with the mouse model, this study strongly supports a role for *ARHGAP35* in vertebrate ocular development, consistent with its known regulation of Hippo-YAP signaling. C-terminal clustering of the identified alleles indicates a possible common mechanism for ocular disease. Other variable developmental features were present in several patients indicating a possible role for *ARHGAP35* in other systems, consistent with the mouse model. Given its known role as a tumor suppressor, further investigation is needed to determine whether individuals with ocular phenotypes are at increased risk for cancer.

## Supplementary information

Supplementary Figure 1

## Data Availability

*ARHGAP35* variants were submitted to ClinVar (Accession numbers SCV002605061 - SCV002605064). There are no additional data available.
