# Supplementary material for: ARHGAP35 is a novel factor disrupted in human developmental eye phenotypes
Source: Eur J Hum Genet. 2022 Dec 1;31(3):363–7. doi: 10.1038/s41431-022-01246-z (PMC9995503; doi:10.1038/s41431-022-01246-z)
Supplement: Supplementary file 1 — Supplementary Figure 1 [file 41431_2022_1246_MOESM1_ESM.pdf]

Supplementary Figure 1: Pedigrees and *ARHGAP35* variant details. A-D. Pedigrees of Families 1-4 with *ARHGHAP35* genotype indicated for all affected individuals and sequence traces/BAM files shown for proband and both parents. Solid symbol indicates affected individual; asterisk indicates presence of variant; WT indicates wild type sequence at position of variant.

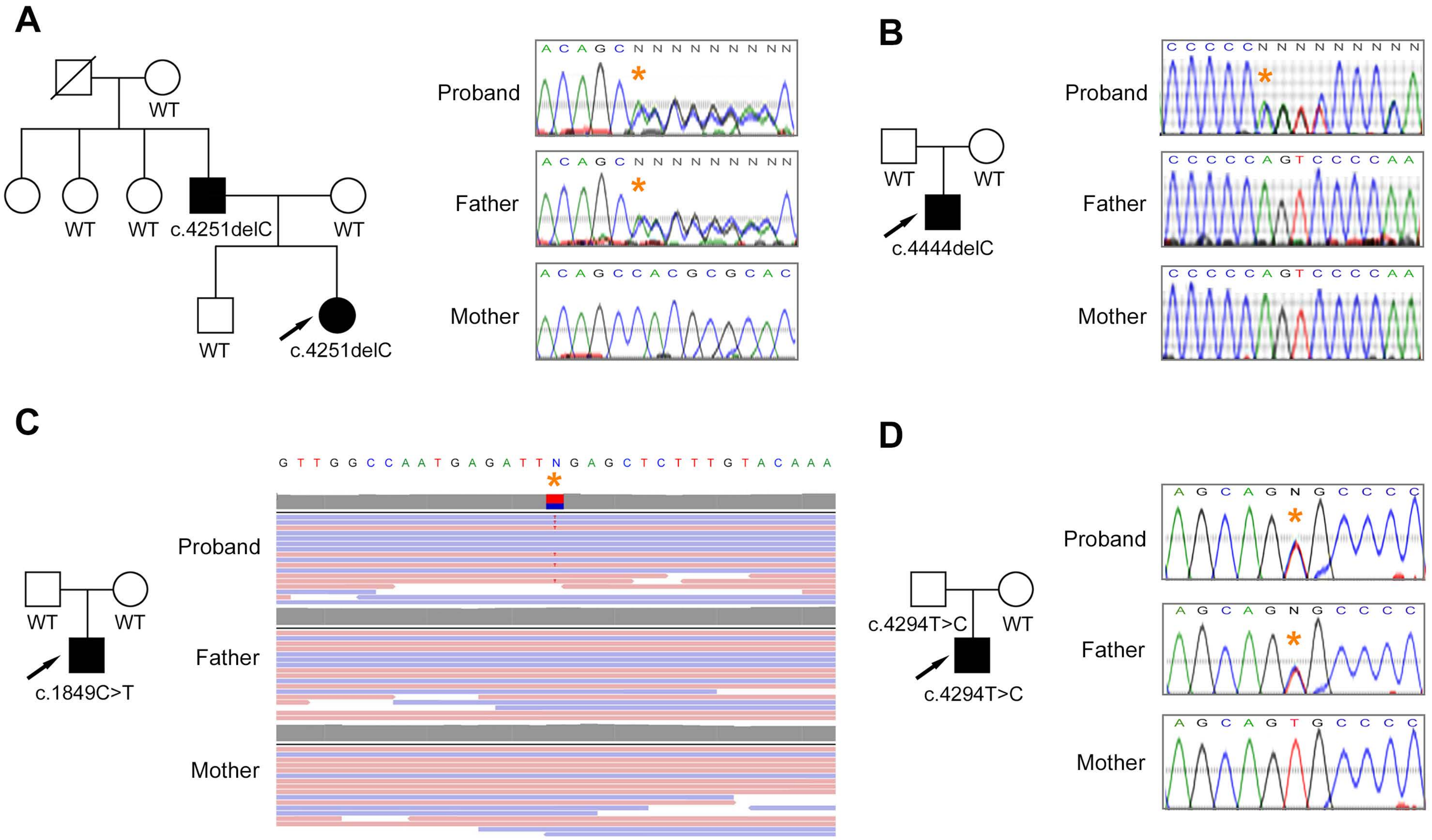
A
WT
WT
WT
c.4251delC
WT
WT
c.4251delC
Proband
Father
Mother
A C A G C N N N N N N N N N
A C A G C C A C G C G C A C
B
WT
WT
c.4444delC
Proband
Father
Mother
C C C C C N N N N N N N N N
C C C C C A G T C C C C A A
C
WT
WT
c.1849C>T
Proband
Father
Mother
G T T G G C C A A T G A G A T T N G A G C T C T T T G T A C A A A
D
c.4294T>C
WT
c.4294T>C
Proband
Father
Mother
A G C A G N G C C C C
A G C A G T G C C C C
